# LDH, CRP and ALB predict nucleic acid turn negative within 14 days in symptomatic patients with COVID-19

**DOI:** 10.1177/0036933021994243

**Published:** 2021-03-04

**Authors:** Gaoli Liu, Bicheng Zhang, Shaowen Zhang, Haifeng Hu, TingTing Liu

**Affiliations:** 1 Attending Physician, Department of Thoracic Surgery, Renmin Hospital of Wuhan University, PR China; 2 Associate Chief Physician, Department of Oncology, Renmin Hospital of Wuhan University, PR China; 3 Attending Physician, Department of Cardiac Function, Renmin Hospital of Wuhan University, PR China

**Keywords:** COVID-19, lactate dehydrogenase, c-reactive protein, procalcitonin, AST, quarantine

## Abstract

**Aims:**

To search for biochemical indicators that can identify symptomatic patients with COVID-19 whose nucleic acid could turn negative within 14 days, and assess the prognostic value of these biochemical indicators in patients with COVID-19.

**Patients and methods:**

We collected the clinical data of patients with COVID-19 admitted to our hospital, by using logistic regression analysis and AUC curves, explored the relationship between biochemical indicators and nucleic acid positive duration, the severity of COVID-19, and hospital stay respectively.

**Results:**

A total of two hundred and thirty-three patients with COVID-19 were enrolled in the study. We found patients whose nucleic acid turned negative within 14 days had lower LDH, CRP and higher ALB (*P* < 0.05). ROC curve results indicated that lower LDH, TP, CRP and higher ALB predicted the nucleic acid of patients turned negative within 14 days with statistical significance(*P* < 0.05), AST, LDH, CRP and PCT predicted the severe COVID-19 with statistical significance, and CRP predicted hospital stay >31days with statistical significance (*P* < 0.05). After verification, the probability of nucleic acid turning negative within 14 days in patients with low LDH (<256 U/L), CRP (<44.5 mg/L) and high ALB (>35.8 g/L) was about 4 times higher than that in patients with high LDH, CRP and low ALB (*P* < 0.05).

**Conclusions:**

LDH, CRP and ALB are useful prognostic marker for predicting nucleic acid turn negative within 14 days in symptomatic patients with COVID-19.

## Introduction

In January 2020, Chinese scientists identified a new type of coronavirus, namely the 2019 new coronavirus (2019-nCoV), thus revealing this infectious viral pneumonia (COVID-19) that had attracted widespread attention.^1^ According to Johns Hopkins data, there were 4,98,22,464 laboratory-confirmed cases worldwide, as of Nov 7, 2020, and 1,246982 cases of death, the death rate was 2.5%.^2^ Global medical resources are bearing a huge burden. Therefore, in order to make rational use of resources, clinicians should identify the patients whose nucleic acid can turn negative in a short time and the severity of disease will be more serious early.

As we known, COVID-19 is an infectious disease. Linton and her college recommended that the length of quarantine in patients with COVID-19 should be at least 14 days.^3^ Lauer reported that under conservative assumptions, 101 out of every 10,000 cases with confirmed SARS-CoV-2 infection outside Hubei province, China would develop symptoms after 14 days of active surveillance or isolation.^4^ But, the characteristics of symptomatic patients who were able to recover within 14 days of isolation have not been reported. COVID-19 is also an acute inflammatory process, with the aggravation of COVID-19, biochemical indicators in patient’s body changed.^5^ Laboratory results indicated that lactate dehydrogenase (LDH) and c-reactive protein (CRP) may be elevated in patients with COVID-19, and procalcitonin (PCT) may be normal.^6^ Previous literatures had suggested that AST, LDH, CRP, and PCT were associated with the prognosis of some inflammatory diseases.^7–12^ However, Whether these makers can identify the patients whose nucleic acid could turn negative during the quarantine (14 days) has not been reported.

In present study, we retrospectively analyzed the clinical data of patients with COVID-19 admitted to our hospital between January 30, 2020 to March 10, 2020, explored the characteristics of biochemical indicators in patients whose nucleic acid turned negative within 14 days, and the relationship between these indicators and the prognosis of patients with COVID-19.

## Patients and methods

### Participants

This study continuously enrolled a series of patients with COVID-19 who were hospitalized in the East Campus, Renmin Hospital of Wuhan University from January 30, 2020 to March 10, 2020. Inclusion criteria for this study were: (1) confirmed COVID-19 cases, (2) hospital stay>14 days, (3) nasopharyngeal-swab test been performed more than twice within 14 days, and^4^ the interval time > 24 hours. Exclusion criteria for this study were: (1) patients with extremely severe COVID-19, (2) patients with liver and/or kidney dysfunction, et al, (3) patients with inflammatory diseases such as chronic obstructive pulmonary disease, pancreatitis, prostatitis, immunological diseases, (4) patients with cerebral infarction or cardiovascular at the time of admission, or (5) patients with missing data.

According to the sixth edition of the COVID-19 Diagnosis and Treatment Guidelines, the diagnostic criteria of COVID-19 were as follows:^13^ (1) A history of contacting with COVID-19 patients; (2) Had fever, cough or gastrointestinal tract discomfort and other clinical symptoms; (3) The results of blood routine examination showed a decrease in leukocyte and/or lymphocyte counts; and (4) In the early stage of the patient's disease, chest CT showed multiple ground-glass shadows or interstitial changes. In the later stage, external lesions appeared in both lungs, presenting invasive changes and even lung consolidation. (5) RT-PCR: the nasopharyngeal-swab specimen of COVID-19 nucleic acid was positive.

COVID-19 was classified as mild, normal, severe, and extremely severe.^13^ Thus, mild COVID-19: symptoms were mild and generally normal on imaging. Common COVID-19 patients may have fever, respiratory or other symptoms, and imaging findings indicate pneumonia. The diagnostic criteria for severe COVID-19 were as follows: (1) shortness of breath, RP ≥30 times/min; (2) in the resting state, peripheral oxygen(SpO_2_) < 93%, partial pressure of oxygen in arteries (PaO2)/fraction of inspiration O2 (FiO2) < 300 mmHg; or (3) pulmonary imaging showed lesion progression > 50% within 24–48 hours. Extremely severe COVID-19 required one of the following conditions: (1) advanced respiratory failure requires ventilator treatment; (2) shock; (3) patients with other organ failure need ICU care.

A confirmed COVID-19 patient requires both 2019n-cov open reading frame 1ab (2019n-cov ORF1ab) and 2019n-cov nucleocapsid protein gene (2019n-cov N gene) to be positive at the same time. The standard of the patient's nucleic acid turned negative was twice consecutive negatives, and the interval of tests > 24 hours.

As our hospital was a government-designated facility for COVID-19, all patients admitted were referred from community hospitals with symptoms. Patients were treated with oral antiviral drugs before admission, and patients with hypertension and/or diabetes routinely received antihypertensive and/or hypoglycemic treatment before and after admission.

Laboratory examination was performed within the first 24 h after admission. We evaluated the severity of the illness during the patient's treatment and collected these parameters from medical records following discharge. CRP was calculated as 5 mg/L if it < 5 mg/L.

### Ethics statement

The treatment of each patient conformed to the ethical principles outlined in the Declaration of Helsinki, and the study protocol was reviewed and approved by the ethics committee of Renmin hospital of Wuhan University (WDRY2020-K059). We also obtained informed consent from the patient or relatives. The data required for the study, including past history, age, sex, BMI and laboratory results were collected after discharge.

### Statistical analysis

Continuous variables in this study were expressed as mean ± standard deviation, and the classification variables were expressed as the number of cases or percentage, as appropriate. We standardized the biochemical indicators as normal score using Blom's Formula. Student's t test, Paired-samples t test or Mann-Whitney U test were used for comparison between two groups, and χ^2^ test for categorical data. The relationship between biochemical indicators and prognosis were assessed by using Pearson or Spearman test, and logistic regression adjusted for age, sex, presence of comorbidities and BMI also was used. We used a receiver operating characteristic (ROC) curve to assess the accuracy of each indicator in predicting prognosis of patients with COVID-19. Data analyses were performed using SPSS 25.0 and MedCalc 18.5. A *P* value <0.05 was considered statistically significant.

## Result

### Patient characteristics

A total of two hundred and thirty-three patients with COVID-19 (105 patients with nucleic acid test turned negative within 14 days, 128 patients with nucleic acid test did not turn negative within 14 days) were enrolled in the study. There were no patients with recurrent COVID-19. Fifteen patients were excluded for the following reasons: one patient with renal failure, two patients with liver dysfunction, ten patients with pulmonary and heart disease, and two patients with acute cerebral infarction at the time of admission. In the present study, there were 42 males and 63 females whose nucleic acid turned negative within 14 days, and the average age was 57.11 ± 13.14 years. The shortest time for nucleic acid to turn negative was 3 days and the longest was 68 days. There were 85 cases accompanied by hypertension or/and diabetes in this study, and 5 of them used ACE2-stimulating drugs before admission.

No statistically differences were observed between the patients whose nucleic acid turned negative within 14 days or not in sex, age > 60 years, the presence of comorbidities, NBMI (Normal Score of body mass index), NALT (Normal Score of Alanine aminotransferase), NGLB (Normal Score of Globulin), NHB (Normal Score of Hemoglobin) (P > 0.05). The patients whose nucleic acid turned negative within 14 days had lower NAST (Normal Score of AST), NLDH (Normal Score of LHD), NTP (Normal Score of Total Protein), NCRP (Normal Score of CRP), NPCT (Normal Score of PCT), higher NALB (Normal Score of Albumin), needed longer hospital stay and had heavier severity than those did not, there were statistically significant (*P* < 0.05) (Table 1).

**Table 1.** Comparison of baseline characteristics among the patients with different time required for nucleic acid to turn negative.

Variables	Nucleic acid turned negative within 14 days	Nucleic acid did not turn negative within 14 days	P value	Statistic
Sex (male)	42	66	0.087	χ^2^ = 3.101
Age > 60 years	48	63	0.597	χ^2^ = 0.315
Comorbidities (Y)	36	49	0.585	χ^2^ = 0.397
NBMI	0.20 ± 1.06	−0.05 ± 0.97	0.063	t = 1.870
NALT	−0.20 ± 1.17	0.06 ± 0.93	0.074	t = −1.795
NAST	−0.23 ± 0.97	0.07 ± 0.99	0.021	t = −2.322
NLDH	−0.35 ± 0.89	0.09 ± 1.00	0.001	t = −3.519
NALB	0.36 ± 1.04	−0.10 ± 0.96	0.001	t = 3.498
NTP	0.26 ± 0.75	−0.07 ± 1.04	0.005	t = 2.830
NGLB	−0.02 ± 0.89	0.01 ± 1.02	0.845	t = −0.195
NCRP	−0.24 ± 0.80	0.11 ± 0.92	0.002	t = −3.061
NHB	0.08 ± 1.11	−0.02 ± 0.96	0.467	t = 0.728
NPCT	−0.27 ± 1.04	0.08 ± 0.95	0.007	t = −2.728
Hospital stay (day)	18.31 ± 8.06	35.63 ± 9.90	0.000	t = −14.717
Severity (1/2/3)	42/54/9	32/85/11	0.044	^χ2^ = 6.256

NBMI: normal score of BMI using Blom's formula; NALT: normal score of ALT using Blom's formula; NAST: normal score of AST using Blom's formula; NLDH: normal score of LDH using Blom's formula; NALB: normal score of ALB using Blom's formula; NTP: normal score of TP using Blom's formula; NGLB: normal score of GLB using Blom's formula; NCRP: normal score of CRP using Blom's formula; NHB: normal score of HB using Blom's formula; NPCT: normal score of PCT using Blom's formula; (Y): (yes); severity (1/2/3): severity (mild/moderate/severe).

In addition, compared with the time of admission, ALT, AST, LDH and CRP decreased, ALB and TP increased after nucleic acid turned negative (P < 0.05) (Table 2).

**Table 2.** The comparison of inflammatory markers at admission and after nucleic acid turned negative.

Variables	At admission	After nucleic acid test turned negative	P value	Statistic
ALT（U/L）	39.19 ± 37.29	36.06 ± 54.15	0.043	Z = −2.023
AST（U/L）	30.72 ± 19.98	28.03 ± 49.83	0.000	Z = −3.888
LDH（U/L）	266.62 ± 98.32	202.87 ± 55.23	0.000	Z = −6.859
ALB（g/L）	36.62 ± 3.98	39.32 ± 3.53	0.000	Z = −5.824
TP（g/L）	61.65 ± 5.05	64.02 ± 5.26	0.000	t = −3.964
GLB（g/L）	25.07 ± 4.54	24.53 ± 4.60	0.105	Z = −1.621
CRP（mg/L）	34.06 ± 40.67	8.61 ± 13.76	0.000	Z = −7.566
HB（g/L）	125.28 ± 16.36	119.74 ± 19.75	0.994	t = −0.008
PCT（ng/ml）	0.08 ± 0.16	0.14 ± 0.68	0.539	Z = −0.614

### The relationship between biochemical indicators and outcomes of prognosis

We analysed the correlation between biochemical indicators and whether nucleic acid turned negative within 14 days, the severity of patients, and hospital stay > 31 days or not, respectively. It revealed that LDH, ALB, TP and CRP were correlated with whether nucleic acid turned negative within 14 days, AST, LDH, ALB, CRP and PCT were correlated with severity of COVID-19, and ALB and CRP was correlated with hospital stay > 31days or not (*P* < 0.05) (Table 3).

**Table 3.** Correlation analysis between inflammatory markers and outcomes of prognosis.

	Nucleic acid turned negative within 14 days or not		Severity of COVID-19		Hospital stay	
Variables	P value	Correlation coefficient	P value	Correlation coefficient	P value	Correlation coefficient
ALT	0.188	0.104	0.451	0.059	0.596	0.042
AST	0.056	0.150	0.002	0.244	0.279	0.085
LDH	0.008	0.207	0.000	0.390	0.055	0.151
ALB	0.008	−0.206	0.010	−0.200	0.015	−0.191
TP	0.044	−0.158	0.377	−0.070	0.054	0.151
GLB	0.910	0.009	0.263	0.088	0.769	0.023
CRP	0.043	0.159	0.000	0.320	0.012	0.196
HB	0.605	−0.041	0.368	0.071	0.730	0.027
PCT	0.073	0.141	0.000	0.407	0.373	0.070

Adjusted for age, sex, BMI and comorbidities, the logistic regression models revealed that patients whose nucleic acid turned negative within 14 days had lower LDH, CRP and higher ALB, patients with heavier severity COVID-19 had higher AST, LDH, CRP, PCT and lower ALB, and with hospital stay > 31 days had a lower CRP(P < 0.05) (Table 4).

**Table 4.** ORs of inflammatory markers for predicting nucleic acid test turned negative within 14 days or not and prognosis of patients with COVID-19.

Factors	Nucleic acid test turned negative within 14 days		Severity of COVID-19		Hospital stay > 31days	
	OR (95% CI)	P Value	OR (95% CI)	P Value	OR (95% CI)	P Value
ALT	1.254 (0.842–1.868)	0.266	1.171 (0.844–1.625)	0.345	1.157 (0.834–1.606)	0.383
AST	1.346 (0.908–1.995)	0.139	1.789 (1.276–2.507)	0.001	1.122 (0.816–1.542)	0.479
LDH	1.709 (1.114–2.624)	0.014	2.965 (2.003–4.388)	0.000	0.925 (0.668–1.280)	0.637
ALB	0.609 (0.398–0.932)	0.022	0.538 (0.375–0.772)	0.001	1.235 (0.879–1.735)	0.224
TP	0.691 (0.462–1.033)	0.072	0.788 (0.566–1.098)	0.160	1.313 (0.941–1.832)	0.109
GLB	0.992 (0.671–1.466)	0.967	1.180 (0.849–1.638)	0.325	1.004 (0.724–1.391)	0.982
CRP	1.602 (1.105–2.580)	0.032	2.391 (1.599–3.574)	0.000	0.611 (0.419–0.891)	0.011
HB	0.866 (0.569–1.320)	0.504	1.076 (0.766–1.512)	0.673	1.002 (0.713–1.408)	0.992
PCT	1.480 (0.985–2.224)	0.059	2.708 (1.855–3.954)	0.000	0.864 (0.625–1.195)	0.378

Adjusted for age, sex, BMI and comorbidities.

### Predictive value of biochemical indicators for whether nucleic acid turned negative within 14 days

We described the ROC curve of **biochemical indicators** for predicting clinical outcomes. For all patients with COVID-19, Lower LDH (cut-off: 256, sensitivity 80%, specificity 50%), TP (cut-off: 61.8, sensitivity 71.4%, specificity 51.6%), CRP (cut-off: 44.5, sensitivity 88.6%, specificity 34.4%) and higher ALB (cut-off: 35.8, sensitivity 77.1%, specificity 50%) predicted nucleic acid turned negative within 14 days with statistical significance, higher AST (cut-off: 28, sensitivity 47.9%, specificity 71.7%), LDH (cut-off: 270, sensitivity 47.0%, specificity 86.96%), CRP (cut-off: 26.8, sensitivity 51.3%, specificity 82.6%) and PCT (cut-off: 0.03, sensitivity 77.8%, specificity 60.9%) predicted severe COVID-19 with statistical significance, combining the indicators of ALT, AST and BMI also could predict severe& extremely severe COVID-19 (*P* < 0.05, sensitivity 62.4%, specificity 78.3%), And higher CRP predicted hospital stay > 31days with statistical significance (cut-off: 46.6, sensitivity 40%, specificity 85.9%) (*P* < 0.05) (Table 5).

**Table 5.** ROC curves in predicting nucleic acid test turned negative within 14 days or not and prognosis of patients with COVID-19.

Factors	Nucleic acid test turned negative within 14 days		Severe COVID-19s		Hospital stay > 31days	
	AUC (95% CI)	P Value	AUC (95% CI)	P Value	AUC (95% CI)	P Value
ALT	0.573 (0.493–0.650)	0.187	0.525 (0.424–0.626)	0.620	0.542 (0.462–0.620)	0.354
AST	0.606 (0.526–0.681)	0.056	0.620 (0.525–0.714)	0.017	0.519 (0.439–0.598)	0.678
LDH	0.645 (0.567–0.719)	0.009	0.697 (0.610–0.785)	0.000	0.541 (0.452–0.630)	0.366
ALB	0.645 (0.540–0.750)	0.009	0.448 (0.358–0.537)	0.298	0.577 (0.497–0.654)	0.091
TP	0.611 (0.514–0.708)	0.044	0.502 (0.406–0.599)	0.966	0.575 (0.495–0.652)	0.099
GLB	0.506 (0.427–0.585)	0.910	0.530 (0.433–0.628)	0.548	0.519 (0.429–0.609)	0.673
CRP	0.610 (0.530–0.685)	0.047	0.659 (0.566–0.752)	0.002	0.621 (0.535–0.707)	0.008
HB	0.529 (0.412–0.645)	0.604	0.541 (0.448–0.634)	0.413	0.517 (0.437–0.596)	0.712
PCT	0.601 (0.521–0.677)	0.073	0.702 (0.609–0.794)	0.000	0.539 (0.450–0.628)	0.386
ALT+AST+BMI			0.762 (0.689–0.825)	0.000		

The AUCs of LDH, ALB, TP and CRP showed no significant difference in predicting nucleic acid turned negative within 14 days(*P* > 0.05) (Table 6).

**Table 6.** Comparison of AUCs in predicting nucleic acid turned negative within 14 days.

Markers	LDH		ALB		TP	
	z statistic	P value	z statistic	P value	z statistic	P value
ALB	0.011	0.991	–	–	–	–
TP	0.558	0.577	0.642	0.521	–	–
CRP	0.783	0.434	0.597	0.551	0.020	0.984

To verify whether the nucleic acid of the patients turned negative within 14 days, we divided the patients into two groups with the cut-off value of **biochemical indicators** as the boundary respectively. The results showed that patients with low LDH (<256 U/L) have a 4-fold higher probability of nucleic acid turning negative within 14 days than those with high LDH(>256 U/L), patients with low CRP(<44.5 mg/L) have a 4.06-fold higher probability than those with high CRP (>44.5 mg/L) and high ALB (>35.8 g/L) was 3.375-fold, there were significant difference (*P* < 0.05) (Table 7).

**Table 7.** RR of inflammatory markers in verifying whether nucleic acid of the patients turned negative within 14 days.

Markers	P value	Statistic	RR(95% CI)
LDH (cut-off:256)	0.002	10.061	4.000 (1.630–9.817)
ALB (cut-off:35.8)	0.007	8.211	3.375 (1.426–7.989)
CRP (cut-off:44.5)	0.011	6.966	4.060 (1.347–12.236)

## Discussion

In this study, we retrospectively analyzed the clinical data of symptomatic patients with COVID-19, logistic regression analysis revealed patients whose nucleic acid turned negative within 14 days had lower LDH, CRP and higher ALB. The ROC curve results indicated that lower LDH, TP, CRP and higher ALB could predicted patients with COVID-19 whose nucleic acid would turn negative within 14 days. After verification, the probability of negative nucleic acid reversion within 14 days in patients with low LDH (<256 U/L), CRP (<44.5 mg/L) and high ALB (>35.8 g/L) was about 4 times higher than that in patients with high LDH, CRP and low ALB. Therefore, our findings suggest that the LDH, ALB and CRP are of practical value in identifying patients with COVID-19 whose nucleic acid could turn negative during the quarantine period (14 days). Meantime, AST, LDH, CRP and PCT are useful prognostic marker for COVID-19.

CRP is an acute phase protein secreted by liver cells during the inflammatory response.^14^ Sun and his colleagues’ study showed CRP was negatively correlated with S-IgG, while S-specific antibodies blocked the S protein's binding to hACE2, a cellular receptor that mediated SARS-COV-2 binding and entering target cells.^15^ In our study, CRP was correlated with whether nucleic acid turned negative within 14 days, the severity of COVID-19, and hospital stay > 31days or not. LDH had been reported as a well-known marker of inflammation and a predictor of various types of pneumonia.^16–18^ Its explanation may be that the distribution of LDH in kidney, heart, liver, lung and other tissues is significantly higher than that in blood, and the changes of LDH induced by these tissues were more obvious.^19^ The research of Yuan et al showed that COVID-19 mRNA clearance ratio was significantly correlated with the decline in LDH levels.^20^ Our study showed lower LDH(<256 U/L) also could identify the patients whose nucleic acid test would turn negative within 14 days. In addition, higher ALB levels (>35.8 g/L) the same function, which may be related to the nutritional status of patients, and more studies are needed to confirm it. PCT can be used as a marker for bacterial infection and may be more effective than other commonly used clinical indicators such as CRP, ESR, and WBC.^21–23^ Our research showed higher PCT had relationship with the severity of COVID-19.

Studies had shown that patients with multiple myeloma with high AST levels had poorer prognosis than those with low AST levels.^24^ However, higher AST as a prediction of poor clinical outcomes is still controversial. Chen and his colleagues found that elevated AST levels in NSCLC patients were closely related to longer relapse-free survival and overall survival.^25^ According to a report in the literature the AST can be expressed in brain, muscle, kidney and other organizations, but ALT is more considered to be liver-specific.^26^ Abnormalities in liver function indicators were more common in patients with COVID-19, however, liver dysfunction may not have serious clinical consequences for patients with COVID-19.^27^ Histological examination of COVID-19 revealed diffuse alveolar damage, lung cell detachment and hyaline membrane formation from lung samples, while atypical lung cell proliferation, and the aggravation of it tends to increase AST.^28^ In this study, higher AST indicated heavier severity of COVID-19, but The ALT changes were not statistically significant. This is consistent with what Mo and her colleagues found.^29^

## Conclusion

The present study shows that LDH(<256 U/L), CRP(>44.5 mg/L) and ALB (>35.8 g/L) could identify the patients with COVID-19 whose nucleic acid could turn negative within 14 days, and AST, LDH, CRP and PCT have the prognostic value for COVID-19. In this way, we can judge which patients can be cured in a shorter period of time, and medical resources can be inclined to those are more complicated.

## Limitations

This study has several limitations. First, this study was a retrospective analysis, and the level of evidence was low. Second, this is a single-center study, which cannot truly reflect the proportion of patients whose nucleic acids can turn negative in 14 days. Although these limitations existed, this study has areas of strength. This is the first study to investigate whether nucleic acids can turn negative in 14 days, which can provide more basis for the health sector to deploy medical resources.
